# Prognosis of pediatric restrictive cardiomyopathy: more severe in sarcomeric variants

**DOI:** 10.3389/fgene.2025.1659218

**Published:** 2026-02-26

**Authors:** Catherine Gardin, Pierre-Emmanuel Michels, Elena Panaioli, Elise Daire, Flavie Ader, Sophie Malekzadeh-Milani, Damien Bonnet, Diala Khraiche

**Affiliations:** 1 Pediatric Cardiology and Pediatric Intensive Care Unit, Angers University Hospital, Angers, France; 2 Psychiatric Department, Angers University Hospital, Angers, France; 3 M3C-Necker, Congenital and Pediatric Cardiology Department, Necker Hospital-Enfants Malades University Hospital, Paris, France; 4 Pediatric Cardiology Department, Amiens University Hospital & Laboratory EA4666 Hematim, University of Picardie–Jules Verne, Amiens, France; 5 Sorbonne Université-DMU BioGem-Unité Fonctionnelle de Cardiogénétique et Myogénétique Moléculaire et cellulaire, Service de Biochimie Métabolique, APHP-Hôpital Universitaire Pitié Salpêtrière, Paris, France; 6 INSERM UMRS1166 Équipe 1, ICAN Institute, Paris, France; 7 Université Paris Cité, UFR de Pharmacie, Paris, France

**Keywords:** cardiomyopathy, heart transplantation, pediatric, restrictive cardiomyopathy, sarcomeric

## Abstract

Restrictive cardiomyopathy (RCM) is the most severe type of cardiomyopathy in children with a very poor prognosis. RCM is often diagnosed between 6 and 10 years old and is predominantly of genetic origin. We conducted a retrospective study of 53 patients. The aim of our study was to determine whether outcomes are associated with the type of genetic variant. We compared the prognosis of patients with sarcomeric variants (n = 26) to those with non sarcomeric variants (n = 27). Our results showed no significant differences between the two groups at diagnosis in terms of heart failure symptoms, NT-proBNP levels, or hemodynamic parameters. However, survival without transplantation was significantly worse in the sarcomeric group (p = 0.003), which also exhibited greater disease severity. Furthermore, thrombotic events were more frequent in the sarcomeric group (p = 0.05). In conclusion, RCM caused by sarcomeric variants is associated with a poorer prognosis and a higher incidence of thrombotic events compared to non-sarcomeric RCM.

## 1 Introduction

In children, **Restrictive cardiomyopathy (RCM)** is defined as the classical form of heart failure with preserved ejectional function. It is characterized by diastolic dysfunction without ventricular dilation, but with significant atrial enlargement. RCM is typically diagnosed between the ages of 6 and 10 years, although rare infantile forms have been reported. It is a rare disease, with an estimated incidence of approximately 0.4 per 100,000 children. Clinical and genetic presentations are heterogenous. (Rath and Weintraub, 2021). The prognosis is poor, with a 5-year mortality rate up to 30%.

In children, the etiology of RCM is predominantly genetic, with pathogenic variants identified in 50%–90% of cases, most often following an autosomal dominant pattern of inheritance. Sporadic *de novo* mutations are frequent, while autosomal recessive, X-linked, or mitochondrial inheritance patterns are rare (Denfield, 2021; Ader et al., 2025).

The main genes implicated in pediatric RCM can be classified in two type: sarcomeric variants and non sarcomeric variants. The biomolecular mechanisms differ fundamentally depending on whether the disease is sarcomeric or non-sarcomeric. Mutations in sarcomeric genes directly alter the mechano-chemical properties of the myofilaments, particularly calcium sensitivity and actin–myosin cross-bridge kinetics, leading to increased passive and active sarcomeric tension, impaired relaxation, and intrinsic diastolic stiffness without major early structural remodeling. In contrast, non-sarcomeric forms primarily result from defects in cardiomyocyte architecture, mechanotransduction, or proteostasis. They are characterized by disruption of the cytoskeletal and sarcomere–costamere network, impaired autophagy, proteotoxic stress, and mitochondrial energetic failure, which secondarily promote interstitial fibrosis, low-grade inflammation, and extrinsic myocardial stiffening.

Thus, sarcomeric forms produce a primary myofilament-driven diastolic dysfunction, whereas non-sarcomeric restrictive cardiomyopathies reflect heart failure dominated by structural, energetic, and protein-quality control abnormalities. In the light of these different mechanisms, we hypothesize that the clinical presentation and the outcome between these two groups might differ.

The aim of our study was to analyze the impact of the type of genetic variant on the clinical course of the disease, based on the presence of a sarcomeric *versus* a non-sarcomeric variant.

## 2 Materials and methods

### 2.1 Patient recruitment and data collection

We conducted a **monocentric retrospective study** including all patients diagnosed with restrictive cardiomyopathy (RCM) after the year 2000 and followed in our institution. RCM was defined according to the European Society of Cardiology as restrictive left and/or Right Ventricle pathophysiology in the presence of normal or reduced diastolic volumes (Of one or both ventricles), normal or reduced systolic volumes, and normal ventricular wall thickness.

We included patients with a positive genetic test, defined by the presence of a pathogenic variant classified as class 4 or 5 according to ACMG guidelines.

Patients were classified based on their genetic variant. The first group included patients with a **sarcomeric variant (SV)**, while the second group included those with a **non-sarcomeric variant (NSV)**. Patients whose histological examination suspected a sarcomeric or non-sarcomeric origin in the absence of genetic data were excluded.

Patients without genetic diagnosis were considered as **unclassified RCM** and excluded from the main study. Patients with an unclear or incomplete clinical diagnosis were also excluded, as were those with RCM secondary to systemic disease or exposure to chemotherapy. We did a comparison between our patients and this unclassified group to assess if differences exist between both.

For each patient, we collected the following data:

Demographic information, including familial history of cardiomyopathy and consanguinity; Echocardiographic and electrophysiological data at diagnosis, as well as biological data, primarily BNP levels; Genetic findings, including the type of variant (when confirmed), details of the variant if available, homozygosity status, and the presence of associated metabolic or syndromic disease; Clinical events (stroke, thrombosis, rhythmic) and their date of occurrence, if known, Therapeutic interventions, including medication, pacemaker and/or defibrillator indications and implantation dates; Cardiac catheterization data, including the date of the procedure and the assessment of pulmonary pressures and vascular resistance; Clinical, echocardiographic, and biological data collected at the time of the last reported evaluation; Date of last evaluation, follow-up duration (in days), date of death, and/or date of heart transplantation.

The last evaluation was defined as the last echocardiographic examination performed before either transplantation or death, or the last evaluation registered in the database. There was no loss to follow-up in our cohort.

All patients were classified according to cardiomyopathy registry standards as having either pure RCM or RCM/HCM/DCM overlap phenotypes. Echocardiographic diagnostic criteria for RCM included biatrial enlargement, normal or reduced ventricular size, and evidence of impaired diastolic function in the absence of significant valvular disease.

We included three phenotypes of RCM (Webber et al., 2012):

Pure RCM, defined by isolated atrial enlargement and diastolic dysfunction without hypertrophic or dilated features; RHCM phenotype, defined by restrictive physiology associated with mild left ventricular hypertrophy; Restrictive-dilated cardiomyopathy (RDCM), defined by restrictive physiology associated with mild left ventricular dilatation (Lipshultz et al., 2019).

### 2.2 Statistical analysis

We considered results statistically significant when the p-value was equal or less than 0.05. Considering this statistical cut off, we paid special attention to results between 0.05 and 0.09 as interesting tendencies.

Statistical analyses were performed using R version 3.5.1 and RStudio software.

Quantitative variables were expressed as median and interquartile range (IQR). Comparisons, including median ages, were done using Fisher’s exact test, due to the small sample sizes.

Qualitative and categorical variables were presented as counts and percentages and compared using the Chi-squared test.

For analyses involving multiple subgroups, multiple regression was employed.

Survival curves were generated using the Kaplan-Meier method.

We also performed a multivariable Cox proportional hazards model to determine independent factors of poor outcomes (death or heart transplantation). We chose six variables for the model: sarcomeric variants, NYHA score, age at diagnosis, gender, arrhythmia, NtproBNP levels at diagnosis. All the proportional hazard tests were above the alpha risk (0.05) allowing the Cox model to be relevant.

Statistical tests were performed with limited subgroups samples. Statistical power remains limited and our results should be evaluated carefully. Moreover, this study is both retrospective and exploratory and p values are considered as indicative of a statistically significant association but cannot be used as *de facto* proof of association.

### 2.3 Ethical statement

This retrospective study was approved by our local ethical committee.

We do not have any conflicts of interest to declare.

## 3 Results

### 3.1 Population characteristics

Eighty-nine patients with restrictive cardiomyopathy were registered. Fifty-three patients were included in our study according to inclusion criteria; they were born between 1 January 1987, and 1 June 2023. All patients underwent at least one clinical and echocardiographic examination at our institution. Thirty-six patients did not have a genetic diagnosis, mainly those diagnosed before 2010. One patient had a negative genetic test.

**FIGURE 1:**
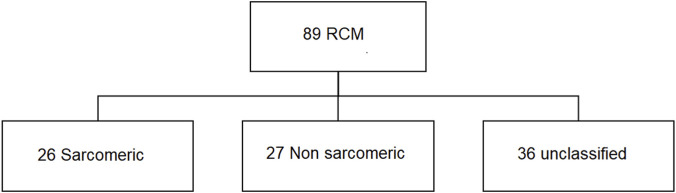
Flow chart.

The flow chart is presented in Figure 1.

In the entire cohort, the mean age at diagnosis was 5.5 years.

Patient characteristics are summarized in Table 1. Extracardiac symptoms reported in the non-sarcomeric group are detailed in Table 2.

**TABLE 1:** Population characteristics at diagnosis.

General characteristics	Sarcomeric (N = 26)	NS (N = 27)	Total (N = 53)	P value
Gender (N, %)	​	​	​	**0.07**
Female (N, %)	17 (65.4)	11 (40)	28 (52.8)	​
Male (N, %)	9 (34.6)	16 (59.2)	25 (47.2)	​
Age at diagnosis (in years)	6.4 (2.3; 11.6)	5.3 (2.5; 11.6)	5.5 (2; 11.8)	0.96
First degree related case (N, %)	8 (30.8)	5 (18.5)	13 (24.5)	0.30
Second degree related case (N, %)	1 (3.8)	4 (14.8)	5 (9.4)	0.17
Antenatal symptoms (N, %)	3 (11.5)	1 (3.7)	4 (7.5)	0.28
Inbreeding (N, %)	7 (26.9)	3 (11.1)	10 (18.9)	**0.05**
Extra cardiac symptoms (N, %)	2 (7.7)	12 (44.4)	14 (26.4)	**< 0.001**
Echographic type at diagnosis (N, %)		​	​	0.14
RCM	18 (69.2)	12 (44.4)	30 (56.6)	​
RHCM	8 (30.8)	14 (51.8)	22 (41.5)	​
RDCM	0 (0)	1 (3.7)	1 (1.9)	​
*De novo* variant	14 (53.8)	22 (81.5)	36 (67.9)	0.01

Bold values are results considered as statistically relevant for our discussion.

Qualitative data are expressed with percentages N (%). Quantitative data are expressed with median and quartiles (Q1-Q3). RCM, Restrictive Cardiomyopathy; RHCM, Restrictive and Hypertrophic Cardiomyopathy; RDCM. Restrictive and Dilated Cardiomyopathy.

**TABLE 2:** Extra cardiac symptoms reported among patients with non sarcomeric variant of interest.

Non sarcomeric mutation	Extra cardiac symptoms
FLNC	Growth retardation, osteochondral disease suspected
Mitochondrial cytopathy	Vascularitis, deafness, interstitial lung disease
BAG 3	Myofibrillar myopathy, axonal neuropathy
Glycogenosis	Low muscle tone
Desminopathy	Food intake disorder, myopathy

In the sarcomeric group, one patient had congenital ichthyosis due to a different genetic cause. Another one, born prematurely at 32 weeks’ gestation, had bronchopulmonary dysplasia. No patients were diagnosed with a specific syndromic disease.

Four patients exhibited antenatal symptoms of cardiomyopathy, mainly fibroelastosis.

In our cohort, consanguinity was significantly more frequent in the sarcomeric group, whereas extracardiac symptoms were mostly reported in the non-sarcomeric group. The occurrence of *de novo* variants was significantly higher in the non-sarcomeric group.

The sex ratio differed slightly, with a higher proportion of female patients in the sarcomeric group.

Regarding other data at diagnosis (antenatal symptoms, first or second-degree related cases), our statistical tests did not permit to show statistical differences between our two groups.

### 3.2 Genetic findings

Genetic testing was performed using targeted next-generation sequencing (NGS) panels or whole-exome sequencing, depending on the date of diagnosis and clinical context.

**FIGURE 2:**
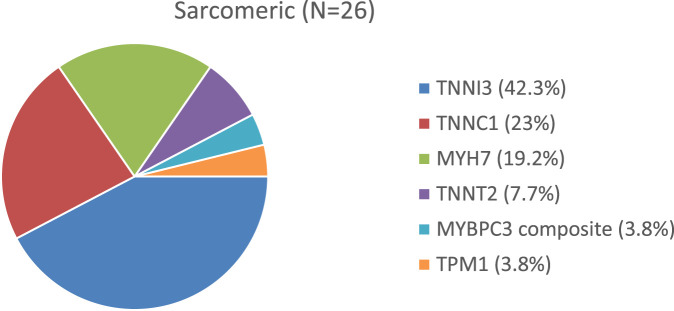
Distribution of genetic variants in the sarcomeric group.

**FIGURE 3:**
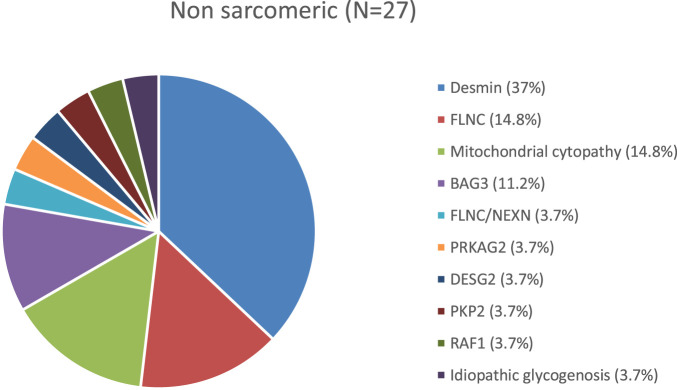
Distribution of genetic variants in the non sarcomeric group.

Two histograms depict the distribution of genetic variants within our two groups (Figures 2, 3).

#### 3.2.1 Sarcomeric group

Fourteen out of 26 cases were related to a *de novo* pathogenic variant: 8 in *TNNI3*, 2 in *MYH7*, 2 in *TNNT2*, 1 in *TPM1*, and 1 in *TNNC1*.

One patient carried a compound heterozygous mutation in *MYBPC3*.

Three cases had inherited heterozygous mutations: 2 in *TNNI3* and 1 in *MYH7*.

Eight cases were associated with homozygous mutations: 1 in *TNNI3*, 3 in *MYH7* (including one patient also harboring a variant of uncertain significance [VUS] in *ACTN2*), and 4 siblings with a homozygous mutation in *TNNC1* (all also carrying a VUS in *MYH6*).

#### 3.2.2 Non sarcomeric group

In the non-sarcomeric group, various genetic mutations were identified.

Twenty-two out of 27 cases were associated with *de novo* mutations: 8 in *DES* (*desmin*), 3 in *BAG3*, 1 in *FLNC*, 1 *NEXN/FLNC* compound heterozygote, 1 in *PRKAG2*, 1 in *RAF1*, and 1 case of idiopathic glycogen storage disease.

Four patients were diagnosed with mitochondrial cytopathy: three involving complex IV and one involving complex II of the respiratory chain. Mitochondrial cytopathies were diagnosed based on findings from endomyocardial and skeletal muscle biopsies with genetic testing.

Five cases had inherited heterozygous mutations: 3 in *FLNC* and 2 in *DES*.

One case was linked to a homozygous mutation in *PKP2*.

### 3.3 Clinical and echocardiographic data at diagnosis

Clinical characteristics at diagnosis are summarized in Table 3.

**TABLE 3:** Clinical data at diagnosis.

Clinical data at diagnosis	Sarcomeric (N = 26)	NS (N = 27)	Total (N = 53)	P value
Asymptomatic (N, %)	6 (23.1)	6 (22.2)	12 (22.6)	0.24
Chest pain (N, %)	6 (23.1)	3 (11.1)	9 (16.9)	0.33
Dyspnea (N, %)	14 (53.8)	15 (55.5)	29 (54.7)	0.33
NYHA score	​	​	​	**0.04**
Grade I - II	5 (19.2)	11 (40.7)	16 (30.2)	​
Grade III - IV	9 (34.6)	4 (14.8)	13 (24.5)	​
Palpitations (N, %)	4 (15.4)	0 (0)	4 (7.5)	**0.07**
Syncope (N, %)	3 (11.5)	2 (7.4)	5 (9.4)	0.33
Hepatomegaly (N, %)	11 (42.3)	10 (37)	21 (39.6)	0.36
Ascitis (N, %)	2 (7.7)	2 (7.4)	4 (7.5)	0.37
NTproBNP (pg/mL) (M, IIQ)	2,842 (1994; 3,315)	1,110 (856; 2,900)	2,556 (1,042; 3,308)	0.71

Bold values are results considered as statistically relevant for our discussion.

Qualitative data are expressed with percentages N (%). Quantitative data are expressed with median and quartiles (Q1-Q3).

Twelve patients (22.6%) were asymptomatic at diagnosis. Among them:

2 had a prenatal diagnosis,

4 were diagnosed through familial screening,

2 were referred for a systolic murmur leading to incidental diagnosis and

4 underwent echocardiography due to non-cardiac symptoms.

Considering the NYHA score, sarcomeric patients were more frequently reported with a severe dyspnea (p = 0.04).

Cardiac imaging and ECG findings are summarized in Table 4.

**TABLE 4:** Cardiologic data at diagnosis.

Cardiac data at diagnosis	Sarcomeric (N = 26)	NS (N = 27)	Total (N = 53)	P value
Abnormal ECG (N, %)	19 (73.1)	22 (81.5)	41 (77.3)	0.28
Arrhythmia (N, %)	6 (23.1)	2 (7.4)	8 (15.1)	0.11
Conduction abnormality (N, %)	1 (3.8)	7 (25.9)	8 (15.1)	**0.02**
Ejection fraction in % (M, IIQ)	65 (58; 70)	65 (62; 70)	65 (59; 70)	0.70
IVSDD in mm (M, IIQ)	9 (8; 13)	9 (7; 11)	9 (7.7; 12.2)	0.70
Z-score IVSDD (M, IIQ)	+3 (+2; +8.8)	+2.6 (+1.8; +4.5)	+2.8 (+1.8; +6.5)	0.14
LVEDD in mm (M, IIQ)	43 (22.7; 43)	36 (28; 39)	36 (25.7; 43)	0.61
Z-score LVEDD (M, IIQ)	+1.1 (−1.7; +1.8)	−0.9 (−2.2; +0.6)	−0.8 (−2.1; +1.6)	0.49
E/A (M, IIQ)	1.4 (0.8; 1.8)	3.2 (2.9; 3.6)	2 (1.3; 3.2)	**< 0.001**
E/E' (M, IIQ)	7 (5.2; 8.6)	10.4 (7; 11.8)	8.1 (5.9; 10.7)	0.34
E' in cm/Sec (M, IIQ)	5 (5; 8.3)	11.5 (10.5; 12.5)	9.5 (5; 12.1)	**0.02**
PAH (N, %)	13 (50)	12 (44.4)	25 (47.2)	0.88
PA mean pressure (M, IIQ)	25 (19; 25)	22.5 (17; 32)	25 (17; 31)	0.63
PA systolic pressure (M, IIQ)	48 (31; 50)	40 (32; 50)	42 (31; 50)	0.70
Bi-atrial enlargement (N, %)	18 (69.2)	19 (70.4)	37 (69.8)	0.96
LAS in cm^2^ (M, IIQ)	19 (13.4; 30)	16 (13.2; 24)	18.5 (13.2; 27)	0.60
LAD in mm (M, IIQ)	36 (33; 41)	40 (37; 45)	40 (34; 44)	0.47
Z-score LAD (M, IIQ)	+5.4 (+3.8; +8.3)	+4.6 (+4.3; +6.7)	+4.8 (+3.9; +8.9)	0.65
VOG/m^2^ (M, IIQ)	54.1 (33; 67.9)	45 (35.5; 57.5)	50.1 (34.2; 63.9)	0.52
ITV in cm (M, IIQ)	14 (12; 18)	15.5 (15; 16)	14.5 (12; 17.5)	0.72
S' wave (M, IIQ)	9 (7; 11)	12 (8; 13)	9.5 (7.5; 12.3)	0.55
TAPSE (M, IIQ)	13 (11; 14)	15 (14.2; 18.7)	14 (12.5; 15)	**0.09**
RV FAC (M, IIQ)	43 (42; 44)	51 (37; 53)	45 (41; 51)	0.98
Mitral regurgitation (N, %)	6 (20)	9 (33.3)	15 (26.3)	0.70
Pericardial effusion (N, %)	3 (10)	2 (7.4)	5 (8.8)	0.81

Bold values are results considered as statistically relevant for our discussion.

Qualitative data are expressed with percentages N (%). Quantitative data are expressed with median and quartiles (Q1-Q3). ECG, Electrocardiogram; IVSDD, Inter-Ventricular Septum Diastolic Diameter; LVEDD, Left Ventricular End Diastolic Diameter; PAH, Pulmonary Artery Hypertension; PA, Pulmonary Artery; LAS, Left Atria Surface; LAD, Left Atria Diameter; RV, Right Ventricle.

#### 3.3.1 ECG findings

The ECG was abnormal at baseline in 77% of the patients, showing abnormal repolarization and signs of atrial and/or left ventricular hypertrophy.

Six patients with sarcomeric mutations were diagnosed with acute arrhythmia. Three were admitted in emergency because of ventricular fibrillation. One had atrial fibrillation. One had supraventricular tachycardia. The precise diagnosis of the arrhythmia could not be done in one patient.

One patient with sarcomeric variant presented conduction abnormality at diagnosis: second degree atrioventricular block.

Among the non sarcomeric group, two patients had rhythmic abnormalities at diagnosis:

1 patient presented atrial fibrillation,

1 presented ventricular fibrillation.

Seven patients presented conduction disorders:

3 had complete atrioventricular block leading to pacemaker implantation,

1 had first degree atrioventricular block, 2 second degree atrio-ventricular blocks,

1 had left bundle branch block.

Despite the small sample size, conduction abnormalities appeared to be more frequent in the non sarcomeric group (p = 0.02).

#### 3.3.2 Cardiac imaging findings

Left atrial enlargement was present in all cases, biatrial dilation was present in 69.8%.

E/A ratio and E′ wave velocity were significantly lower in the sarcomeric group (p < 0.05).

No other echocardiographic differences between groups reached statistical significance.

### 3.4 Catheterism

Hemodynamic data are presented in Table 5.

**TABLE 5:** Data at the first catheterization procedure.

Catheterism	Sarcomeric (N = 15)	NS (N = 14)	Total (N = 29)	P value
Time to first catheterization in days (M, IIQ)	155 (32; 222)	187 (59; 315)	181 (24; 254)	0.25
PA systolic pressure (M, IIQ)	40 (36; 50)	44 (29; 55)	40 (32; 50)	0.57
PA mean pressure (M, IIQ)	27 (24; 36)	28 (21; 38)	27 (23; 36)	0.78
Capillary pressure (M, IIQ)	17 (14; 20)	20 (14; 25)	18 (14; 20)	0.41
Indexed PVR (M, IIQ)	5 (2.8; 5.8)	3.1 (3; 12)	4.5 (2.9; 6.7)	0.19
Indexed Cardiac Output (M, IIQ)	2.6 (2.2; 3.1)	1.9 (1.4; 2.4)	2.3 (1.8; 2.6)	0.17
Central venous pressure (M, IIQ)	11 (7; 12)	10 (8; 14)	11 (7; 12)	0.53

Quantitative data are expressed with median and quartiles (Q1-Q3). PA, Pulmonary Artery; PVR, Pulmonary Vascular Resistance (Wood Units); PVR and cardiac output were indexed on corporeal surface. Pressures are expressed in mmHg and cardiac output in L/min/m^2^.

Twenty-four patients did not undergo cardiac catheterization. In most cases, the procedure was performed as part of the pre-transplantation evaluation.

There were no significant differences between the sarcomeric and non-sarcomeric groups regarding hemodynamic parameters obtained during catheterization.

### 3.5 Survival and outcomes

#### 3.5.1 Survival

Outcomes are reported in Table 6.

**TABLE 6:** Outcomes and follow-up.

Outcomes	Sarcomeric (N = 26)	NS (N = 27)	Total (N = 53)	P value
Heart transplantation (N, %)	12 (46.1)	7 (25.9)	18 (33.9)	**0.07**
Age at HT in years (M, IIQ)	10.6 (3.2; 13.4)	12.2 (6.6; 15.9)	10.6 (4.4; 13.9)	0.29
Time to transplant (M, IIQ)	8.8 (7.1; 18.6)	66.8 (27.2; 104.3)	22.2 (8.2; 27.3)	**0.003**
Death (N, %)	9 (34.6)	14 (51.9)	23 (47.4)	0.21
Age at death in years (M, IIQ)	1.5 (1.1; 5.3)	5.4 (1; 12.8)	3 (1; 7.7)	0.10
Time to death (M, IIQ)	8.7 (3.1; 18.3)	12.1 (11.7; 60.4)	10.9 (3.9; 18.3)	0.15
Death etiology (N, %)	​	​	​	**0.05**
Sudden death	4 (15.4)	0 (0)	4 (7.5)	​
VF/VT	2 (7.7)	1 (3.7)	3 (5.7)	​
Thrombo-embolic, Stroke	1 (3.8)	0 (0)	1 (1.9)	​
Heart failure	2 (7.7)	9 (33.3)	11 (20.7)	​
Last NTproBNP (M, IIQ)	4,630 (2,250; 5,090)	1,385 (937; 1741)	2,250 (1,500; 4,767)	**0.004**
Follow-up duration (M, IIQ)	239 (107; 664)	741 (241; 2034)	349 (108; 1,425)	**0.09**

Bold values are results considered as statistically relevant for our discussion.

NTproBNP measurement are expressed in ng/L.

Qualitative data are expressed with percentages N (%). Quantitative data are expressed with median and quartiles (Q1-Q3). HT, Heart Transplantation; VF, Ventricular Fibrillation; VT, Ventricular Tachycardia; Time to transplant and time to death are counted in months.

Time to heart transplantation was significantly shorter in the sarcomeric group (p = 0.003).

NT-proBNP levels at the last available examination were also significantly higher in this group.

**FIGURE 4:**
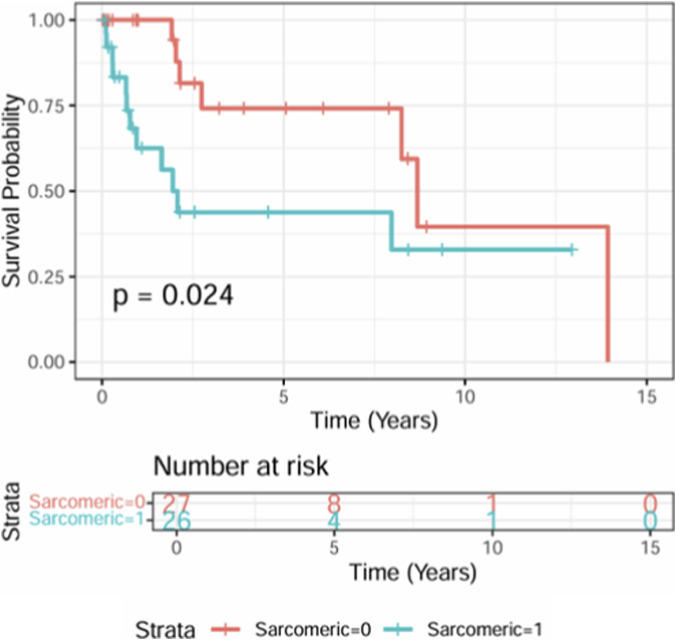
Kaplan-Meier analysis considering time free from transplantation.

The Kaplan-Meier survival curve (Figure 4) represents the transplant-free survival for both groups. The delay to heart transplantation was significantly shorter in patients with sarcomeric mutations (p < 0.01).

Among deceased patients, aetiologies are slightly different, with an occurrence of sudden death higher in the sarcomeric group and more cardiac insufficiency in the non sarcomeric group (p = 0.05).

The results of our multivariate Cox proportional hazards model is resumed in Table 7.

**TABLE 7:** Multivariable Cox model analysis.

Covariates	HR (CI 95%)	p
Death model	​	​
Sarcomeric variant	0.64 (0.18; 2.26)	0.49
Age at diagnosis >5 years old	**0.07 (0.02; 0.44)**	**0.003**
Gender	2.6 (0.71; 9.38)	0.14
NYHA score 2	**12.6 (2.05; 77.2)**	**0.006**
NYHA score 3	3.73 (0.69; 20)	0.12
NYHA score 4	NA	NA
NtproBNP level at diagnosis >2000 ng/mL	0.51 (0.04; 6.51)	0.61
Arrhythmia	**10.09 (1.21; 83.3)**	**0.03**
Heart transplant model	​	​
Sarcomeric variant	25.5 (0.24; 2,727)	0.17
Age at diagnosis >5 years old	0.46 (0.02; 8.39)	0.61
Gender	0.14 (0.005; 3.95)	0.25
NYHA score 2	0.59 (0.0005; 666.8)	0.89
NYHA score 3	4.41 (0.03; 150.9)	0.56
NYHA score 4	NA	NA
NtproBNP level at diagnosis >2000 ng/mL	0.66 (0.002; 150.9)	0.88
Arrhythmia	1.84 (0.04; 81)	0.75

Bold values are results considered as statistically relevant for our discussion.

HR, Hazard ratio; CI, confidence interval.

Patients with a NYHA score 2 are associated with a greater risk of death compared to NYHA 1 as patients with arrhythmia. Being younger than 5 years old at diagnosis is associated with a higher risk of death. There was no statistical difference considering other covariates.

All covariates were not associated with a greater risk of heart transplantation in this model.

#### 3.5.2 Complications

Long term complications are reported in Table 8.

**TABLE 8:** Long term complications.

Complications	Sarcomeric (N = 26)	NS (N = 27)	Total (N = 53)	P value
Rythmic events and conduction disorders	​	​	​	​
Abnormal holter (N, %)	2 (7.7)	4 (14.8)	6 (11.3)	0.11
High grade atrioventricular block (N, %)	1 (3.8)	4 (14.8)	5 (8.7)	0.36
Arrhythmia (N, %)	9 (34.6)	6 (22.2)	19 (26)	0.24
Supra-ventricular arrhythmia (N, %)	5 (19.2)	4 (14.8)	9 (16.9)	​
Ventricular arrhythmia (N, %)	4 (15.4)	2 (7.4)	6 (11.3)	​
Time to first rhytmic event, months (M, IIQ)	2 (0.2; 29.6)	14 (4.7; 36)	5.3 (0.8; 36)	0.74
Pacemaker (N, %)	0 (0)	4 (14.8)	4 (7.5)	**0.04**
Implanted ICD (N, %)	3 (11.5)	1 (3.7)	4 (7.5)	0.28
Thrombotic events	​	​	​	​
Intra cardiac thrombosis (N, %)	3 (11.5)	0 (0)	3 (5.7)	**0.05**
Stroke (N, %)	3 (11.5)	0 (0)	3 (5.7)	0.07
Antiplatelets (N, %)	1 (3.8)	7 (25.9)	8 (15.1)	**0.04**
Anticoagulation (N, %)	12 (46.1)	5 (18.5)	17 (32.1)	0.07

Bold values are results considered as statistically relevant for our discussion.

Qualitative data are expressed with percentages N (%). Quantitative data are expressed with median and quartiles (Q1-Q3). ICD, Intracardiac cardioverter defibrillator.

Thrombotic events were significantly more frequent in the sarcomeric group (p = 0.05) and the occurrence of strokes was marginally higher (p = 0.07).

Anticoagulation therapy was also frequently prescribed in this group and was initiated either when pre-thrombotic signs were observed on echocardiography, or when a thrombus was diagnosed. Thus, the number of patients with anticoagulation therapy was slightly higher in the sarcomeric group (p = 0.07).

Two patients without sarcomeric mutations were diagnosed with rhythmic disorder during follow-up:

1 had episodes of ectopic atrial tachycardia, and 1 presented episode of repeated ventricular extra-systoles.

Pacemaker implantations were done only in the non-sarcomeric group, with a significantly higher proportion compared to the sarcomeric group (p = 0.04).

Concerning the occurrence of conduction disorders and arrhythmia, there was no statistical differences between the two groups.

#### 3.5.3 Supplementary analysis: comparison with the unclassified patients

Patients excluded from the study because of missing genetic diagnosis were gathered in the unclassified group. After conducting the main analysis, we compared our studied population to the unclassified one, checking for unexpected differences.

This comparison is reported in Table 9.

**TABLE 9:** Characteristics of the unclassified group.

Summarized characteristics	Unclassifled (N = 36)	Studied population (N = 53)	P value
Gender	​	​	0.28
Female (N, %)	24 (66.7)	28 (52.8)	​
Male (N, %)	12 (33.3)	25 (47.2)	​
Total (N, %)	36 (100)	53 (100)	​
Age at diagnosis in years (M, IIQ)	5.2 (1.3; 9.7)	5.5 (2.2; 11.7)	0.59
First degree related case (N, %)	10 (27.8)	13 (24.5)	0.92
Second degree related case (N, %)	0 (0)	5 (9.4)	0.15
Antenatal symptoms (N, %)	2 (5.6)	4 (7.5)	**<0.001**
Inbreeding (N, %)	7 (19.4)	10 (18.9)	0.34
Extra cardiac symptoms (N, %)	6 (16.7)	14 (26.4)	0.41
Echocardiographic type at diagnosis	​	​	**0.07**
RCM	27 (75)	30 (56.6)	​
RHCM	7 (19.4)	22 (41.5)	​
RDCM	2 (5.6)	1 (1.9)	​
Cardiac data at diagnosis	​	​	​
Bi-atrial enlargement (N, %)	31 (81.6)	37 (69.8)	0.13
E/A (M, IIQ)	1.9 (1.3; 2)	1.9 (1.2; 3.2)	0.28
Outcomes and adverse events	​	​	​
Thombosis (N, %)	2 (5.6)	3 (5.7)	0.92
Stroke (N, %)	1 (2.8)	3 (5.7)	0.19
High grade atrioventricular block (N, %)	1 (2.8)	4 (7.5)	0.21
Arrhythmia (N, %)	11 (30.5)	19 (35.8)	0.99
Pacemaker (N, %)	1 (2.8)	4 (7.5)	0.62
Defibrillator (N, %)	3 (8.3)	4 (7.5)	0.99
Heart transplantation (N, %)	7 (19.4)	18 (33.9)	0.21
Age at heart transplant in years (M, IIQ)	13 (7.5; 17.8)	10.6 (4.4; 13.8)	0.36
Death (N, %)	13 (36.1)	23 (43.4)	0.99
Age at death in years (M, IIQ)	5.9 (2.3; 8.4)	3 (1; 7.7)	0.63

Bold values are results considered as statistically relevant for our discussion.

Qualitative data are expressed with percentages N (%). Quantitative data are expressed with median and quartiles (Q1-Q3). RCM, Restrictive cardiomyopathy; RHCM, Restrictive-hypertrophic cardiomyopathy; RDCM, Restrictive dilated cardiomyopathy.

There was no major difference between those two groups excepted a higher rate of prenatal signs of RCM (p < 0.001).

## 4 Discussion

Restrictive Cardiomyopathy (RCM) is the rarest type of cardiomyopathy, accounting for 2.5%–5% of cardiomyopathies in children. Its definition has long lacked consensus (Chintanaphol et al., 2022; Konta et al., 2015). The American Heart Association (AHA) classifies primary cardiomyopathies based on their pathophysiology as genetic, acquired or mixed. (Nihoyannopoulos and Dawson, 2009). In children, most RCM cases are genetic, in contrast to adults, and are sometimes associated with syndromic diseases, storage diseases or muscular dystrophy (Kaski et al., 2024).

RCM is commonly defined as a myocardial disease characterized by impaired ventricular filling of one or both ventricles in the presence of normal systolic function and normal or near-normal myocardial thickness, resulting in reduced diastolic volume.

While the “pure” RCM phenotype is more common (65% of cases (Webber et al., 2012)), some patients exhibit mild left ventricular hypertrophy (RHCM) or mild left ventricular dilatation (RDCM). In our cohort, 41.5% of patients presented with RHCM. Pure RCM forms were more frequently reported in the sarcomeric group.

We encountered limitations in evaluating mixed RHCM phenotypes due to the hypertrophic component. In adolescents, the Z-score for interventricular septal thickness was often elevated (above +7), even when the diastolic IVS measurement was only modest (14–16 mm), reflecting cases of young adults with low body weight. This observation underscores the limitations of Z-scores in evaluating adolescent patients with adult body morphology but classified using pediatric criteria (Pettersen et al., 2008; Cantinotti et al., 2012).

The most frequent mode of inheritance in pediatric RCM is sporadic. Depending on the cohort, genetic variants are identified in 50%–90% of cases, including both sporadic and familial forms (Webber et al., 2012; Ranthe et al., 2015; Denfield and Webber, 2010). In our study, sarcomeric RCM was associated with a higher rate of consanguinity, compared to the non-sarcomeric group. This aligns with prior research demonstrating that familial inheritance increases recurrence risk in offspring. A Danish study from 2015 reported a 6- to 400-fold increased risk of recurrence in the presence of a family history of cardiomyopathy (Ranthe et al., 2015).

The most common genetic variants in inherited RCM affect sarcomeric structural proteins, followed by cytoskeletal proteins (Chintanaphol et al., 2022). Numerous genes have been implicated in familial or sporadic RCM, including cardiac troponin T (*TNNT2*), troponin I (*TNNI3*), myosin-binding protein C (*MYBPC3*), *MYH7*, myosin light chains (*MYL2*, *MYL3*), desmin (*DES*), *MYPN*, titin (*TTN*), *BAG3*, *DCBLD2*, *LMNA*, and *FLNC* (van den Wijngaard et al., 2011; Shah et al., 2017; Gigli et al., 2016; Mogensen et al., 2003). The number of known associated variants continues to grow.

Sarcomeric genes encode components such as actin, myosin, troponin, and titin. Prior studies have identified variants in *MYH7*, *ACTC1*, *TNNI3*, and *TNNT2* as leading causes of RCM (Webber et al., 2012; Yang et al., 2022). Some variants are shared with hypertrophic cardiomyopathy (HCM), reinforcing the hypothesis that RCM and HCM may represent two phenotypic expressions of the same genetic disease (Yang et al., 2022). Restrictive cardiomyopathy with hypertrophic features may serve as a clinical bridge between these two entities.

The genetic spectrum in our cohort aligns with known data: *TNNI3* and *MYH7* were the most frequently identified sarcomeric variants, while *FLNC*, mitochondrial disorders, and desminopathy were more frequent in the non-sarcomeric group (Kostareva et al., 2016).

The gene affected appears to influence the clinical phenotype. Sarcomeric mutations tend to produce isolated cardiac disease (Sato and Ito, 2021), while non-sarcomeric variants often affect multiple organs, making RCM a partial manifestation of broader systemic disease (Lee et al., 2017).

The literature shows a wide variety of phenotypes in cardiomyopathy. The same variant could lead to a neonatal severe disease but also stay unexpressed. This diversity is well described today, and constitutes a trademark of sarcomere-based pathology. But across this inconstancy, identifying variant signatures is the key of future personalized medicine.

The phenotype variability of RCM is correlated to the exact sarcomeric variant. In our cohort, most patients with restrictive-hypertrophic cardiomyopathy were diagnosed with a *TNNI3* variant (5 out of 8 RHCM). All ICD were implanted for patients with RHCM, and 2 out of 3 were also *TNNI3* patients.

Considering rhythmic events, 50% of our sarcomeric patients who presented acute arrhythmia at diagnosis were reported with an *MYH7* variant. But more than 60% of patients with severe arrhythmic events reported in the follow up were assessed with a *TNNI3* variant. Moreover, among the six patients reported with thrombotic events (intracardiac thrombus or stroke), 4 were *TNNI3* patients, and 1 *MYH7* patient.

*MYH7* is one of the most frequently reported variant in pediatric hypertrophic cardiomyopathy. The risk stratification is improving concerning HCM, and it appears the stake is similar in RCM. Our data suggests *MYH7* variant and *TNNI3* variant should be taken very seriously concerning life threatening events in RCM.

In comparison, in the non sarcomeric group, pure RCM echocardiographic form were due to desminopathy in 50% of cases (while desminopathy represents only 27% of the unpure RCM subgroup). 4 out of 7 patients with a conduction abnormality at diagnosis were associated with a desminopathy. 75% of patients with pacemakers were reported with desminopathy.

As in HCM, the genotype variant is probably the cornerstone to help practitioners to identify patients with a higher risk of severe events.

Extra-cardiac symptoms were more common in the non-sarcomeric group, reflecting the multisystem nature of these disorders. Some RCM diagnoses were made based on extra-cardiac features, supporting the notion that the presence of both cardiac and systemic symptoms is more specific to non-sarcomeric forms. These differences also lead to divergent clinical courses (Muchtar et al., 2017).

Our findings indicate that sarcomeric RCM is more severe, with faster disease progression than non-sarcomeric RCM. Patients with a sarcomeric variant were reported with a higher NYHA score, highlighting the severity of the disease. Echocardiographic data showed significantly worse diastolic function in the sarcomeric group. There was a higher rate of heart transplantation, and a shorter time to transplant, among sarcomeric patients (Castiglione et al., 2022; Salem et al., 2022).

Cardiomyopathies are the leading cause of heart transplantation in children over 1 year of age (Wilkinson et al., 2010). RCM is a particularly severe form, with a median transplant-free survival of 2.2 years (Rath and Weintraub, 2021). Given the high risk of developing pulmonary hypertension, early referral for transplantation is recommended (Mori et al., 2022; Denfield et al., 1997). However, in our study, the time to transplant differed significantly between the two groups: The median time to transplant in the sarcomeric group was 8.8 months vs. 66.8 in the non sarcomeric group. Our results highlight the necessity of risk stratification according to the genetic variant. In patients with non sarcomeric variant, early referral for transplant might not be as crucial as in sarcomeric variant and can be based on clinical tolerance of the cardiomyopathy (Author Anonymous, 2017; Anderson et al., 2018).

The severity of the disease is also due to arrhythmias and conduction disorders. A 2018 study reported that almost 60% of children listed for heart transplant due to RCM had arrhythmias and 16% had experienced cardiac arrest (Wittekind et al., 2019). In our cohort, 15.1% had arrhythmias diagnosed at the first consultation (Table 4). Conduction disorders were more frequent in the NS group, necessitating a PM implantation in 4 patients, all in the NS group.

Due to the high risk of thrombotic events, prevention of thrombosis is recommended in RCM.

In our study, 5.7% of patients developed intracardiac thrombi, all within the sarcomeric group. This finding suggests that prophylactic anticoagulation might be appropriate with sarcomeric mutations.

In life-threatening arrhythmia or in patients with a high-risk profile, implantable cardioverter-defibrillators (ICDs) may be considered. However, decision-making should be individualized, as evidence is limited and recommendations for ICDs in RCM are generally extrapolated from HCM guidelines (Writing et al., 2021; Walsh et al., 2012). Despite differences in risk profiles, patients with RCM still face a high risk of sudden cardiac death (Webber et al., 2012; Rivenes et al., 2000).

However, ICD implantation is invasive and carries a high complication rate in children, particularly in younger patients (Le Bos et al., 2023). Given the young age at diagnosis (mean 5.5 years) and short transplant-free survival in sarcomeric cases, the benefit of ICD implantation is debatable. Additionally, our patients often required epicardial systems due to low body weight, necessitating a sternotomy, which could complicate future transplantation.

In conclusion, RCM in children is a severe disease, and prognosis is significantly worse in patients with sarcomeric mutations. Our results support risk stratification based on genetic subtype, advocating for early transplant listing and systematic anticoagulation with sarcomeric RCM.

## 5 Conclusion

Restrictive cardiomyopathy is the most severe type of cardiomyopathy in children. However, sarcomeric mutations define a clinically more severe subtype leading to a rapid transplantation and more thrombotic events. At the opposite, non sarcomeric RCM have a slower disease progression and are characterized by frequent conduction abnormalities and extracardiac symptoms. Knowing the genetic cause of the RCM helps stratify the risk and the management of these patients.

## 6 Limitations

The main issue of our study was the size of our cohort. It is a great limitation regarding statistical power but also interpretation of our results. Moreover, because of the retrospective evaluation, we also faced several missing data.

Our Cox model analysis was limited because of sample size and tendencies may be clearer with bigger samples.

Following the main analysis, we compared our studied population with the unclassified patients. 36 patients were excluded from our study, representing a great number of patients regarding the rarity of the RCM disease.

Among these 36 patients, no genetic diagnosis existed, mostly because of the period of diagnosis, where genetic tests were not so easily available. The number of genes concerned by the specific screening was also lower. These patients represent a significant number of unavailable data, which marred on our global overall results.

4 out of these 36 patients were siblings, with a supposed sarcomeric history of RCM, because of familial history of cardiomyopathy, and histological findings on cardiac tissue.

We expect our results would have been slightly different with 36 supplementary cases. We compared this unknown group with our overall cohort (Table 8). The two population did not differ except for the prenatal diagnosis of RCM, which is consistent with the major evolution of prenatal diagnosis in the recent years.

Thus, we do not have accurate elements to identify the way our results would have been influenced by these missing data. We are still limited by the small number of cases in each group, which includes limited statistical power.

## Data Availability

The datasets presented in this article are not readily available because our database includes dates of birth, date of diagnosis, date of death and date of transplant, which constitute potentially identifiable data that cannot be shared. Requests to access the datasets should be directed to: gardincatherine@orange.fr and diala.khraiche@aphp.fr.
