# Supplementary material for: Prognosis of pediatric restrictive cardiomyopathy: more severe in sarcomeric variants
Source: Front Genet. 2026 Feb 26;16:1659218. doi: 10.3389/fgene.2025.1659218 (PMC12978873; doi:10.3389/fgene.2025.1659218)
Supplement: Supplementary file 1 [file Table1.pdf]

Table 9: Descriptive data for supplemental analysis.

This supplementary table gathers relevant informations in the whole cohort, precising genetic variant, if precisely reported, for each patient.

AET : Atrial Ectopic Tachycardia. AF : Atrial Fibrillation. AIVR : Accelerated Idioventricular Rythm. CH : Compound Heterozygous. HT : Heart Transplant. NA : Non available data. RCM: Restrictive Cardiomyopathy. RHCM: Restrictive and Hypertrophic Cardiomyopathy. RDCM : Restrictive and Dilated Cardiomyopathy. SD : Sudden Death. SVT : Supraventricular tachycardia. UVT : Unsustained Ventricular Tachycardia. VF : Ventricular fibrillation. VT : Ventricular Tachycardia. VUS : Variant of Unknown Significance. YO : Years Old..

PATIENT	MUTATION	REPORTED VARIANT	HOMOZYGOUS	INBREEDING	AGE (YO)	PHENOTYPE	BIATRIAL	ARRHYTHMIA	DEFIBRILLATOR	COMPLETE BLOCK	PACEMAKER	STROKE	THROMBUS	OUTCOM
1	TNNC1 + MYH6 VUS	Exon01,c.23>T,p.Ala8Val/ Exon30, c.4284G>T,p.Glu1428Asp	1	1	NA	RCM	NA	VF	0	0	0	0	0	DEATH
2	TNNC1 + MYH6 VUS	Exon01,c.23>T,p.Ala8Val/ Exon30, c.4284G>T,p.Glu1428Asp	1	1	NA	RCM	NA	SD	0	0	0	0	0	DEATH
3	TNNC1 + MYH6 VUS	Exon01,c.23>T,p.Ala8Val/ Exon30, c.4284G>T,p.Glu1428Asp	1	1	0	RCM	NA	0	0	0	0	0	0	HT
4	TNNC1 + MYH6 VUS	Exon01,c.23>T,p.Ala8Val/ Exon30, c.4284G>T,p.Glu1428Asp	1	1	0.2	RCM	1	SD	0	0	0	0	0	DEATH
5	FLNC	exon41,c.6826G>A,p.Val2276Met	0	0	11.9	RHCM	1	0	0	0	0	0	0	ALIVE
6	Mitochondrial cytopathy complex II	Unavailable	0	0	4.3	RCM	1	0	0	1	1	0	0	DEATH
7	FLNC / NEXN compound heterozygous	Exon25,c.4289-?_c?4927+?del,p.?/Exon09,c.1053+1G>A	0	0	0.8	RHCM	1	0	0	0	0	0	0	DEATH
8	Desminopathy	Unavailable	0	1	0.6	RCM	1	0	0	0	0	0	0	DEATH
9	Desminopathy	Unavailable	0	1	0.7	RCM	1	0	0	0	0	0	0	DEATH
10	TNNI3	Exon08,c.584T>C,p.Ile195thr/Exon248,c.46089_46090del,p.cys15363Trpfs*5	0	0	0.8	RCM	1	0	0	0	0	0	0	HT
11	Desminopathy	Unavailable	0	0	8.8	RHCM	1	0	0	0	0	0	0	HT
12	TNNT2	Exon09,c.280C>G,p.(Arg94Gly)	0	0	2.6	RHCM	0	SVT/VT	0	1	0	0	0	DEATH
13	TNNI3	c.439G>C,p.Val147Leu	1	1	11.6	RHCM	1	VF	1	0	0	0	0	HT
14	Mitochondrial cytopathy complex IV	Unavailable	0	0	1.8	RHCM	1	0	0	0	0	0	0	DEATH
15	Mitochondrial cytopathy complex IV	Unavailable	0	0	14.7	RHCM	1	AF	0	0	0	0	0	DEATH
16	Desminopathy	Unavailable	0	0	1.2	RHCM	NA	VF	1	0	0	0	0	DEATH
17	PRKAG2	Exon15,c.1592G>A,p.(Arg531Gln)	0	0	0	RHCM	1	0	0	0	0	0	0	DEATH
18	TNNC1	Exon03, c.79T>G, p.Phe27Val	0	0	0.1	RCM	1	0	0	0	0	0	0	DEATH
19	DESG2	Exon08, c.1360C>T, p.Arg454Trp/Exon08, c.991G>A, p.Glu331Lys	0	0	9.7	RHCM	0	0	0	0	0	0	0	ALIVE
20	Desminopathy	Unavailable	0	0	7.7	RCM	1	0	0	0	0	0	0	DEATH
21	Desminopathy	Unavailable	0	0	4.8	RCM	1	0	0	0	0	0	0	DEATH
22	TNNI3	c.509G>A, p.Arg170Gln	0	0	8.9	RCM	1	0	0	0	0	0	0	HT
23	MYH7	c.1987C>A, p.Arg663Ser/c.3330dupG, p.Leu1111Alafs*21	1	1	14.8	RHCM	1	AF	0	0	0	0	1	HT
24	Desminopathy	Unavailable	0	0	2.7	RCM	1	0	0	1	1	0	0	HT
25	Desminopathy	Unavailable	0	NA	14.2	RDCM	1	0	0	0	0	0	0	DEATH
26	PKP2	Unavailable	1	1	3.2	RHCM	1	0	0	0	0	0	0	HT
27	RAF1	Exon16, c.1728C>G, p.Asn576Lys	0	0	5.3	RCM	1	AF	0	0	0	0	0	HT
28	TNNI3	Exon07,c.506delT, p.Leu169Argsfs*8	0	0	12.6	RHCM	1	AIVR	0	0	0	0	0	HT
29	TNNI3	Exon 07,c.508C>T, p.Arg170Trp	0	0	5.5	RCM	1	0	0	0	0	1	1	HT
30	Mitochondrial cytopathy complex IV	Unavailable	0	0	5	RCM	1	0	0	0	0	0	0	DEATH
31	Glycogenosis	Unavailable	0	0	0.9	RCM	0	0	0	0	0	0	0	HT
32	TNNI3	Exon07, c.508C>T, p.Arg170Trp	0	0	5	RHCM	1	0	0	0	0	1	0	DEATH
33	TNNI3	Exon08, c.610C>T, p.Arg204Cys	0	0	12.1	RCM	0	0	0	0	0	0	0	HT
34	TPM1	Unavailable	0	0	2.5	RCM	1	0	0	0	0	0	0	HT
35	BAG3	Exon03, c.626C>T, p.Pro209Leu	0	0	13.4	RHCM	1	EAT	0	0	0	0	0	DEATH
36	TNNI3	c.522G>C, p.Lys174Asn	0	0	7.3	RHCM	1	0	0	0	0	1	0	DEATH
37	TNNI3	Unavailable	0	0	2.3	RCM	0	SD	0	0	0	0	1	DEATH
38	BAG3	Unavailable	0	0	8.8	RHCM	0	0	0	0	0	0	0	ALIVE
39	MYH7	Exon09, c.769A>G, p.Lys257Glu	0	0	0	RCM	1	SD	0	0	0	0	0	DEATH
40	FLNC	Exon41,c.6826G>A, p.Val2276Met	0	0	12.1	RHCM	0	0	0	0	0	0	0	ALIVE
41	MYBPC3 compound heterozygous	MYBPC3 c.1927+937G>T et c.1828G>C Hz composite	0	1	16.2	RHCM	1	UVT	1	0	0	0	0	ALIVE
42	MYH7	Exon 21, c.2334C>G, p.Asp778Glu	1	0	16.6	RCM	1	AF	0	0	0	0	0	ALIVE
43	MYH7 + ACTN2 VUS	Exon 35, c.5057C>T, p.Leu1685Pro/ Exon 11, c.1192C>T, p.Arg398Cys	1	0	0.2	RCM	0	0	0	0	0	0	0	ALIVE
44	MYH7	Exon 22, c.2511G>C, p.Lys837Asn	0	0	3.8	RCM	1	0	0	0	0	0	0	ALIVE
45	TNNI3	Exon 07, c.4703 C>T, p.Ala157Val	0	0	7.3	RHCM	0	VT	1	0	0	0	0	ALIVE
46	FLNC	Exon 37, c.6031G>T, p.Gly2011Trp	0	0	3.5	RCM	0	0	0	0	0	0	0	ALIVE
47	BAG3	Unavailable	0	0	11.5	RCM	1	Flutter	0	0	0	0	0	ALIVE
48	Desminopathy	Unavailable	0	0	12.9	RHCM	0	AF	0	1	1	0	0	HT
49	FLNC	EXON 41, c.6958G>A, p.Gly2320Arg	0	0	10.5	RHCM	1	0	0	0	0	0	0	ALIVE
50	Desminopathy	Unavailable	0	0	15	RCM	NA	0	0	1	1	0	0	HT
51	TNNT2	Exon 9 c.280C>T, p.Arg94Cys	0	0	9.8	RCM	1	0	0	0	0	0	0	HT
52	TNNI3	Exon 08, c.575G>A, p.Arg192His	0	0	10.4	RCM	1	0	0	0	0	0	0	HT
53	MYH7	Exon 22 c.2563_2565del	0	0	12.8	RCM	1	VT	0	0	0	0	0	HT
